# Genome-wide identification, characterization, and expression analysis of tea plant autophagy-related genes (*CsARGs*) demonstrates that they play diverse roles during development and under abiotic stress

**DOI:** 10.1186/s12864-021-07419-2

**Published:** 2021-02-17

**Authors:** Huan Wang, Zhaotang Ding, Mengjie Gou, Jianhui Hu, Yu Wang, Lu Wang, Yuchun Wang, Taimei Di, Xinfu Zhang, Xinyuan Hao, Xinchao Wang, Yajun Yang, Wenjun Qian

**Affiliations:** 1 College of Horticulture, Qingdao Agricultural University, Qingdao, 266109 China; 2 National Center for Tea Plant Improvement, Tea Research Institute, Chinese Academy of Agricultural Sciences, Hangzhou, 310008 China; 3 Key Laboratory of Tea Biology and Resources Utilization, Ministry of Agriculture, Hangzhou, 310008 China; 4 College of Agriculture and Food Science, Zhejiang A&F University, Hangzhou, 311300 China

**Keywords:** Autophagy, *Camellia sinensis*, Expression, Hormone, Abiotic stress, Cold acclimation

## Abstract

**Background:**

Autophagy, meaning ‘self-eating’, is required for the degradation and recycling of cytoplasmic constituents under stressful and non-stressful conditions, which helps to maintain cellular homeostasis and delay aging and longevity in eukaryotes. To date, the functions of autophagy have been heavily studied in yeast, mammals and model plants, but few studies have focused on economically important crops, especially tea plants (*Camellia sinensis*). The roles played by autophagy in coping with various environmental stimuli have not been fully elucidated to date. Therefore, investigating the functions of autophagy-related genes in tea plants may help to elucidate the mechanism governing autophagy in response to stresses in woody plants.

**Results:**

In this study, we identified 35 *C. sinensis* autophagy-related genes (*CsARG*s). Each *CsARG* is highly conserved with its homologues from other plant species, except for *CsATG14*. Tissue-specific expression analysis demonstrated that the abundances of *CsARG*s varied across different tissues, but *CsATG8c*/*i* showed a degree of tissue specificity. Under hormone and abiotic stress conditions, most *CsARG*s were upregulated at different time points during the treatment. In addition, the expression levels of 10 *CsARG*s were higher in the cold-resistant cultivar ‘Longjing43’ than in the cold-susceptible cultivar ‘Damianbai’ during the CA period; however, the expression of *CsATG101* showed the opposite tendency.

**Conclusions:**

We performed a comprehensive bioinformatic and physiological analysis of *CsAR*Gs in tea plants, and these results may help to establish a foundation for further research investigating the molecular mechanisms governing autophagy in tea plant growth, development and response to stress. Meanwhile, some *CsARGs* could serve as putative molecular markers for the breeding of cold-resistant tea plants in future research.

**Supplementary Information:**

The online version contains supplementary material available at 10.1186/s12864-021-07419-2.

## Background

Autophagy (ATG) is an evolutionarily conserved eukaryotic system that involves the degradation of various cytoplasmic components, including many biological macromolecules (such as proteins and protein aggregates), and entire organelles in vacuoles or lysosomes [1]. Normally, autophagy occurs at basal levels in eukaryotic cells but induces autophagic flux by specific developmental processes or stressful environments to degrade oxidative damaged proteins, damaged organelles, and other toxic compounds. Autophagy ensures that the degradation products can be recycled and the cells remodeled in cells to sustain their survival. Autophagy occurs through at least four pathways, classified as microautophagy [2], macroautophagy [3], chaperone-mediated autophagy [4], and selective autophagy [5]. Among these pathways, macroautophagy, which is manipulated by a special organelle, known as the autophagosome, is the most thoroughly characterized and is commonly referred to as autophagy. In this report, macroautophagy is referred to as autophagy. Accumulating evidence indicates that macroautophagy is derived from the formation of cup-shaped double membranes named phagophores (or isolation membranes), which engulf cytoplasmic material and then obturate to generate autophagosomes. Next, the outer membrane of the autophagosome fuses with the tonoplast, and the rest of the autophagosome forms an autophagic body, which is degraded in the vacuolar lumen to release its cargo for recycling [6].

Autophagy is primarily mediated by a collection of *ATG* genes. To date, more than 30 *ATG* genes have been identified in yeast and *Arabidopsis* [7–11]. Among these genes, *ATG1–10*, − *12-14*, − *16-18*, − *29*, and − *31* serve as key modulators that participate in autophagy initiation, nucleation, elongation, maturation, and fusion with vacuoles [12–14]. In yeast and *Arabidopsis*, the initiation of autophagy is mediated by the target of rapamycin (TOR) kinase via association with ATG1/13 [15]. Next, the PI3K complex, containing Vps34, Vps15, ATG6/Vps30 and ATG14, is activated to promote vesicle nucleation. This step is followed by the expansion and enclosure of autophagy through the ATG5-12-16 and ATG8-PE conjugation systems. Later, the autophagosome is docked and fused to the tonoplast, which employs a vesicle trafficking system, known as the v-SNARE complex. Finally, the autophagic body in the vacuole is digested by a series of hydrolases, including the lipase ATG15 and proteinases A (PEP4) and B (PRB1) [16]. Following the identification of the ATG protein families in yeast and *Arabidopsis*, numerous orthologs have been identified in various plant and animal genomes, indicating that the core autophagic systems controlled by these proteins are conserved during evolution. In recent years, *ATG* genes have been identified in a number of plant species, such as 33 *OsATGs* in rice [17], 30 *NtATGs* in tobacco [18], 24 *SlATGs* in tomato [19], 30 *VaARGs* in grapevine [20], 32 *MaATGs* in banana [21], 37 *SiATGs* in foxtail millet [22], and 29 *CaATGs* in pepper [23]. Based on the alignment of multiple ATG amino acid sequences, many of the ATGs showed remarkable overall conservation in various plants, strongly suggesting that autophagic processes are mechanistically identical in different plants.

Along with the identification of *ATG* genes across the eukaryotic kingdoms, accumulating evidence indicates that the mechanism governing autophagy is involved in the whole life cycle of the plant, ranging from vegetative and reproductive development to environmental stress responses. Recently, reverse genetics approaches have effectively accelerated the functional analyses of *ATG* genes in plants, especially mutation and overexpression techniques. Many research findings have indicated that most *ATG*-mutated *Arabidopsis* lines exhibit premature senility phenotypes, although they still have intact life cycles, indicating that autophagy contributes to leaf longevity during senescence [9, 11, 24, 25]. Autophagy addresses organ senescence and nutrient starvation (e.g., carbon and nitrogen starvation, sucrose deficiency, and lack of light) by degrading damaged or unwanted proteins and organelle compounds to promote the recycling and remobilization of nutrients [1, 26–29]. It has been demonstrated that the biomass production and nitrogen remobilization efficiency in *ATG*-mutated *Arabidopsis* and rice were notably lower than those in the wild-type plants, indicating that autophagy contributes to efficient nitrogen remobilization [29–31]. In addition, the synthesis of amino acids was reduced in autophagy mutants during carbon starvation, indicating that the autophagy machinery controls cellular homeostasis [32]. However, increased autophagic activity can promote yield and nitrogen use efficiency in plants. For example, overexpression of *OsATG8a* was observed to strongly improve the level of autophagy and significantly improved nitrogen uptake efficiency in transgenic rice under suboptimal N conditions [26]. Apart from responding to nutrient deficiency, autophagy can also be induced by various abiotic stresses, and the kinases SnRK1 and TOR may be the central regulators of these processes [27]. Under high salt and osmotic stress conditions, the expression of an autophagy-related gene, *AtATG18a*, was upregulated in *Arabidopsis*, and the *AtATG18a* mutants were more sensitive to salt and drought conditions than were the wild-type plants [33]. However, overexpression of *ATG5* or *ATG7* promoted Atg8 lipidation, autophagosome formation and autophagic flux, thereby increasing the resistance of necrotrophic pathogens and oxidative stress and delaying senescence and improving growth, seed set, and seed oil content [34]. Moreover, autophagy can shape plant innate immune responses by a variety of means, but there are three main ways to induce autophagy, including virulent or related pathogen-induced programmed cell death, salicylic acid and jasmonic acid, and virus-induced RNA silencing [35]. Overall, autophagy is involved in the regulation of the whole life cycle of plants during different growth phases under different growing conditions.

As a special type of evergreen plant, the tea plant (*Camellia sinensis*) requires specific growing conditions, such as acidic soil, high humidity, and ordinary temperature; thus, this plant is primarily distributed in tropical and subtropical areas in Asia. However, tea plants are also regularly exposed to pathogen attack and herbivory, nutrient deficiency, and various types of abiotic challenges, such as extreme temperature, drought, salt, ozone, and ion toxicity. Under these adverse conditions, the morphology, physiology, and metabolism of tea plants have changed to survive. Accordingly, numerous studies on the stress resistance of tea plants in response to various stimuli have been performed in recent years. For example, multiple omic techniques, including transcriptomic, proteomic and metabonomic techniques, have been widely employed to explore the dynamic changes in genes, proteins and metabolites under different stress conditions [36–40]. In addition, many genes that respond to various stimuli have been identified and analyzed using draft genome sequences [41–46], and functional studies of some differentially expressed genes (DEGs), such as the Basic leucine zipper (bZIP) gene (*CsbZIP6*) [47], SWEET transporter gene (*CsSWEET16*) [48], vacuolar invertase gene (*CsINV5*) [49], and 12-Oxophytodienoate reductase gene (*CsOPR3*) [50], have been widely performed. However, no one autophagy-related gene (*ARG*) has been comprehensively analyzed in tea plants, and the roles played by autophagy in coping with different environmental stimuli have also not been fully elucidated to date in tea plants. In this study, the in vivo roles played by autophagy and the mechanisms governing the expression of *ARG* genes in tea plants were investigated through the genome-wide identification, characterization, and expression analysis of *CsARGs*. The results of this study may facilitate a deeper understanding of the diverse roles played by autophagy in response to different growth phases or environmental stress conditions in tea plants.

## Results

### Identification of *CsARGs* in tea plants

Based on four different identification methods, a total of 35 *CsARGs* were identified from the two published tea plant genomes (‘ShuChaZao’ and ‘YunKang10’). Among these genes, four genes (*CsATG1s*, *CsATG8s*, *CsATG18s* and *CsVTI13s*) were determined to have isoforms (Table 1). In this study, we identified a UV radiation resistance protein/autophagy-related protein 14, known as *CsATG14*, which has not been fully studied in plants to date and may be deficient in *Arabidopsis*. In addition, as there is no single strict criterion to identify all the paralogs of these four genes, and as the genome assembly and gene annotation of the two reported tea plant genomes have not been fully completed to date compared to the *Arabidopsis*, rice and tobacco genomes, the products of the partial paralogs of these four genes, as mentioned in *Arabidopsis*, rice, and tobacco, were not determined in the tea plant genomes. Bioinformatic analysis results showed that as a type of biological macromolecule, the *CsARG* ORF lengths varied from 285 to 7410 bp, the corresponding numbers of deduced amino acids ranged from 94 to 2469 aa, and the molecular weights ranged from 10.51 to 276.87 kD. The theoretical isoelectric points (pIs) were predicted to range from 4.52 to 9.41. The prediction of subcellular location results suggested that most *Cs*ARGs were located in the nucleus, and some of them were also predicted to be located in the cytoplasm, chloroplasts and mitochondria. The results of signal peptide prediction showed that none of these *Cs*ARGs contained signal peptides. In addition, *Cs*ATG9 was predicted to contain 5 TMHs, *Cs*ATG18b, and three transport vesicle-soluble NSF attachment receptor (v-SNARE) proteins, namely, *Cs*VTI12, *Cs*VTI13a and *Cs*VTI13b, were predicted to contain 1 TMH.

**Table 1:** Basic information of CsARGs

Gene name	Accession number	ORF (bp)	AA	MW (kDa)	pI	instability index	Aliphatic index	Loc	SignalP	TMHs
CsATG1c	XP_028071137.1	2193	730	80.78	6.69	unstable	83.37	Nucleus	NO	NO
CsATG1t	XM_028254805.1	852	283	31.70	6.72	unstable	100.21	Cytoplasm	NO	NO
CsATG2	XM_028214951.1	6039	2012	220.59	5.69	unstable	87.78	Nucleus	NO	NO
CsATG3	XM_028269562.1	945	314	35.72	4.72	unstable	78.82	Cytoplasm	NO	NO
CsATG4	XM_028205167.1	1473	490	54.16	5.54	unstable	73.45	Nucleus	NO	NO
CsATG5	XM_028239072.1	1047	367	41.29	4.77	unstable	98.26	Cytoplasm	NO	NO
CsATG6	XM_028209545.1	1581	526	59.43	5.87	unstable	71.77	Nucleus	NO	NO
CsATG7	XM_028207488.1	2121	706	77.95	5.63	unstable	91.20	Cytoplasm	NO	NO
CsATG8a	XM_028204481.1	354	117	13.65	6.60	unstable	84.19	Cytoplasm	NO	NO
CsATG8c	XM_028257959.1	360	119	13.65	8.78	unstable	83.61	Nucleus	NO	NO
CsATG8f	XM_028237334.1	369	122	14.00	8.75	stable	95.08	Nucleus	NO	NO
CsATG8g	XM_028213593.1	354	117	13.64	8.73	stable	86.67	Nucleus	NO	NO
CsATG8i	XM_028202806.1	393	130	14.96	7.58	unstable	64.38	Nucleus	NO	NO
CsATG9	XM_028219288.1	2610	869	99.93	6.25	unstable	79.55	plasmid	NO	5
CsATG10	XM_028214294.1	717	238	27.19	4.96	unstable	82.73	Nucleus	NO	NO
CsATG11	XM_028237709.1	3471	1156	129.98	5.57	unstable	82.95	Nucleus	NO	NO
CsATG12	XM_028206145.1	285	94	10.51	9.41	unstable	88.19	Cytoplasm	NO	NO
CsATG13	XM_028260289.1	1863	620	68.75	8.90	unstable	65.27	Nucleus	NO	NO
CsATG14	XM_028265144.1	1440	479	53.78	8.85	unstable	77.37	Chloroplast	NO	NO
CsATG16	XM_028206301.1	1527	508	55.74	6.08	unstable	91.44	Cytoplasm	NO	NO
CsATG18a	XM_028253213.1	1296	431	47.63	6.62	stable	77.82	Nucleus	NO	NO
CsATG18b	XP_028071781.1	1107	368	40.18	7.15	unstable	96.49	Cytoplasm	NO	1
CsATG18c	XM_028196882.1	1257	418	46.36	8.01	unstable	84.40	Chloroplast	NO	NO
CsATG18f	XM_028238387.1	2697	898	97.13	8.47	unstable	75.46	Mitochondria	NO	NO
CsATG18g	XM_028202982.1	2988	995	108.70	5.78	unstable	80.87	Chloroplast	NO	NO
CsATG18h	XM_028252480.1	3069	1022	111.90	5.79	unstable	76.91	Chloroplast	NO	NO
CsATG20	XM_028265532.1	1206	401	46.15	8.20	unstable	83.47	Chloroplast	NO	NO
CsATG101	XM_028236970.1	657	218	25.43	6.46	stable	88.03	Nucleus	NO	NO
CsATI	XP_028079241.1	948	315	35.00	4.52	unstable	64.13	Nucleus	NO	1
CsVTI12	CSA033576	669	222	25.26	9.22	unstable	105.81	Nucleus	NO	1
CsVTI13a	XM_028240825.1	666	221	25.12	9.30	unstable	102.81	Cytoplasm	NO	1
CsVTI13b	XM_028226760.1	666	221	24.89	9.41	unstable	101.95	Cytoplasm	NO	1
CsVPS15	XM_028202873.1	4632	1543	172.13	6.19	unstable	87.43	Nucleus	NO	NO
CsVPS34	XM_028243914.1	2361	814	93.36	6.39	unstable	92.46	Cytoplasm	NO	NO
CsTOR	XM_028205854.1	7410	2469	276.87	6.22	unstable	102.06	Cytoplasm	NO	NO

*ORF* opening reading fame, *AA* the numbers of amino acid residues, *pI* Theoretical isoelectric point, *MW* Molecule weight, *Loc* Subcellular location, *TMHs* Transmembrane helices

### Phylogenetic analysis of *Cs*ARGs in tea plant

**Fig. 1:**
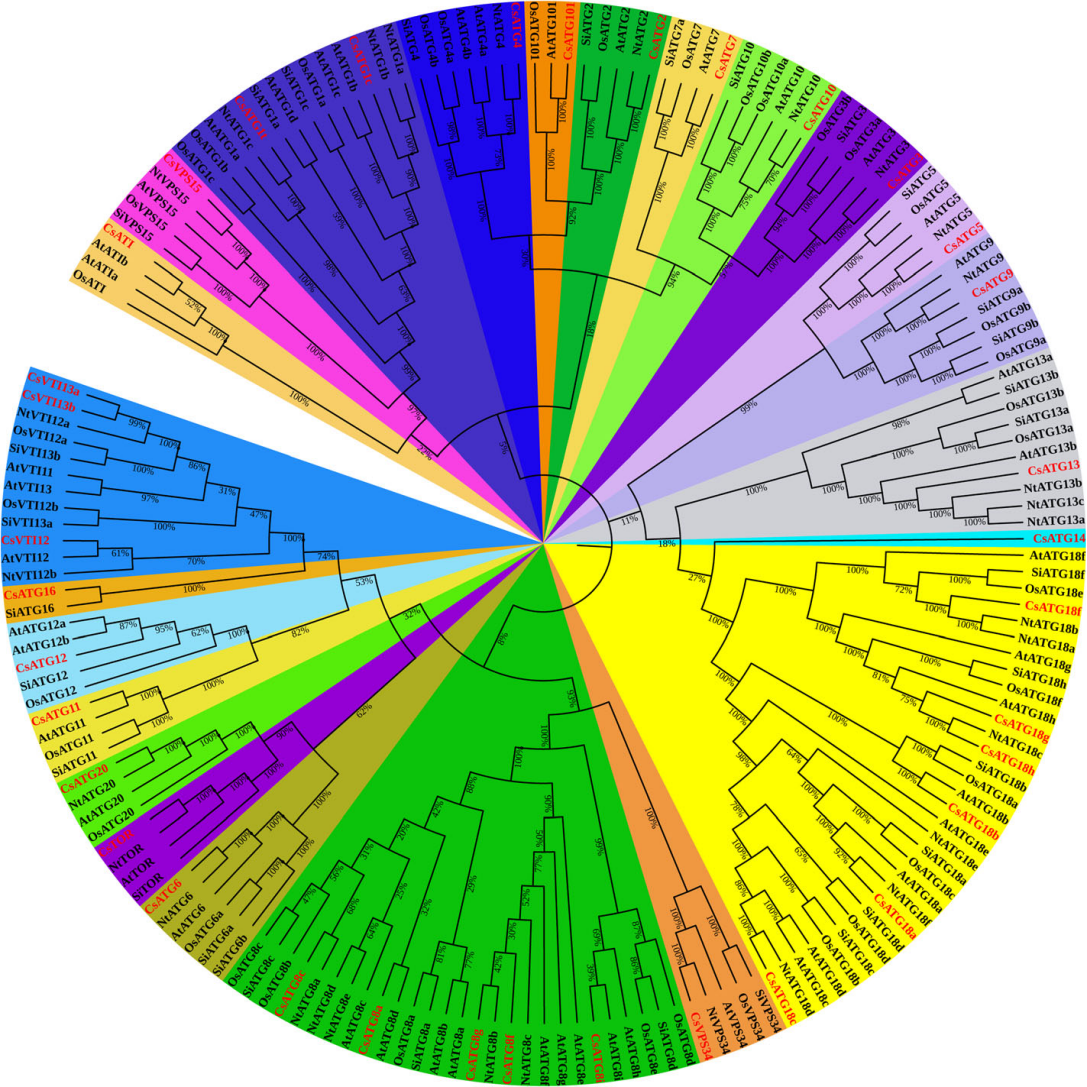
Phylogenetic analysis of CsARGs and known ARGs in *Arabidopsis*, *Setaria italic*, *Oryza sativa* and *Nicotiana tabacum*. A total of 177 ARG protein sequences were used to construct phylogenetic tree throughout the neighbor-joining method with 1000 repeated bootstrap tests, p-distance, and pairwise deletion in MEGA 5.0 software. CsARGs are highlighted with red color, and different ARG subfamilies were covered with different colors

To explore the evolutionary relationships and classification of *Cs*ARGs in tea plant, a total of 177 ARG proteins from tea plants, *Arabidopsis*, *Setaria italic*, *Oryza sativa*, and *Nicotiana tabacum* were aligned to construct a phylogenetic tree. As shown in Fig. 1, except for *Cs*ATG14, which had only been identified in tea plants, all of the *Cs*ARG proteins were highly clustered together with the homologous proteins derived from the other four species, and almost all of the *Cs*ARGs showed the closest relationship with *Nt*ARGs. Meanwhile, we observed that the bootstrap values among the different ARG proteins in each subtree were nearly 100%, except for the ATG8 subfamily, which suggests that ARG protein sequences are highly conserved and may exhibit similar functions among different species.

### Gene structure, protein domain distribution and *cis*-acting element analysis

**Fig. 2:**
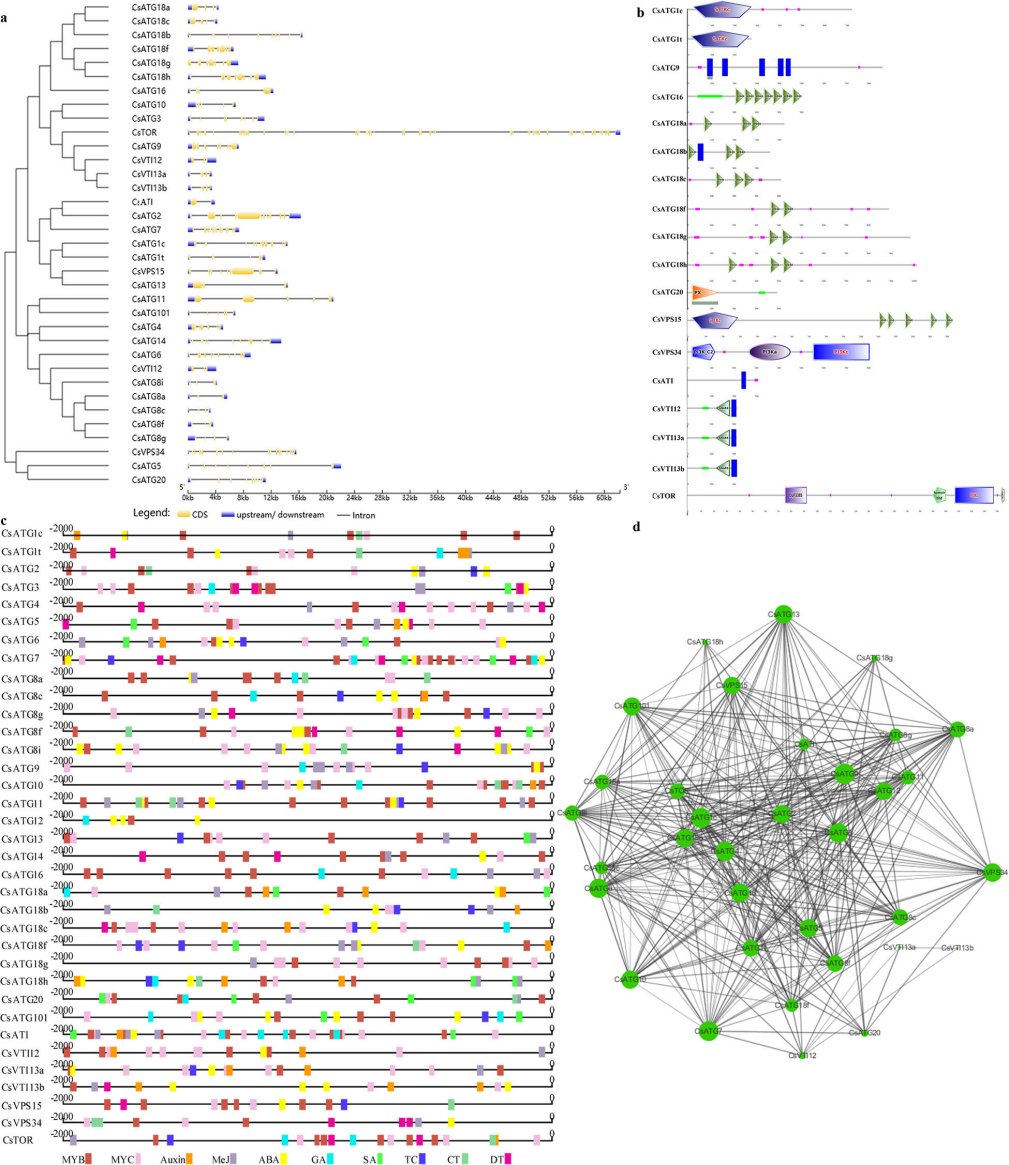
The exon-intron structures, protein domains, *cis*-acting elements and protein-protein interaction networks of CsARGs. **a** Exon-intron structure of *CsATG* genes. The coding sequence and the corresponding genomic sequence of each *CsARG* were compared by using the Gene Structure Display Server (GSDS) program. Blue boxes represent untranslated upstream/downstream regions, yellow boxes represent exons, and lines indicate introns. **b** Protein domains of CsARGs. **c** The *cis*-acting regulatory elements of *CsARGs*. 2000-bp upstream noncoding region sequences of each *CsARG* gene were used to predict *cis*-acting elements, and different colored blocks represent different elements. **d** Protein-protein interaction networks of CsARGs. Thirty five orthologs of CsARGs were obtained from Inparanoid web server, and those 35 orthologs formed 360 protein-protein association patterns

Understanding the exon-intron structure is beneficial for exploring the evolution of multiple gene families [51]. To investigate how the differences in exon-intron structure were generated, both the genomic and ORF sequences of *CsARGs* were uploaded into GSDS v2.0 to predict the exon-intron structure. As shown in Fig. 2a, the numbers of exons in the *CsARG* family varied, with members within the *CsATG8s* or *CsVTI13s* subfamilies exhibiting similar exon-intron structures.

To further dissect the functions of *Cs*ARG proteins, the protein domains of each *Cs*ARG were analyzed by the SMART program. As shown in Fig. 2b, *Cs*ATG1s encode serine/threonine protein kinases, which contain catalytic domains involved in protein phosphorylation occurring during the progression of autophagy [52]. *Cs*ATG9 contains 5 transmembrane helix regions (88–110, 155–177, 320–342, 403–425 and 438–457), as detected by the TMHMM v2.0 program, which play a unique role in autophagosome formation derived from the endoplasmic reticulum (ER) in plants [53]. *Cs*ATG16 contains a coiled coil region and 7 WD40 domains, which form a conserved Atg12-Atg5-Atg16 complex during the autophagy process. Each member of the *Cs*ATG18s subfamily is largely a β-propeller and is formed by 2 or 3 WD40 domains. *Cs*ATG20, also known as Snx42, contains a PX domain, which plays a central role in efficiently inducing nonselective autophagy. *Cs*VPS15 encodes a serine/threonine protein kinase, which is formed by 5 WD40 domains and is regulated by a phosphoinositide 3-kinase (PI3K), *Cs*VPS34. At present, *Cs*VPS34 is characterized as a central regulator in mediating vesicular trafficking and cellular homeostasis [54]. As an ATG8-interacting protein, *Cs*ATI contains a transmembrane region that may help the protein complex to move to the ER network and reach the lytic vacuole. *Cs*VTI1s, including *Cs*VTI12, *Cs*VTI13a and *Cs*VTI13b, all contain a coiled coil region, a t-SNARE domain and a transmembrane region, and these domains may be involved in the trafficking of cargoes to the vacuole. *Cs*TOR, as a conserved phosphatidylinositol kinase-related protein kinase, contains a specific rapamycin-binding domain, a P13Kc-catalyzing domain and a FATC domain, which suggests that it is involved in mediating redox-dependent structural and cellular stability.

To elucidate the regulatory mechanisms governing *CsARG*s in response to growth and development, stress defenses, and hormone signaling, a 2000-bp 5′-upstream noncoding region sequence of each *CsARG* was isolated to predict *cis*-elements. As Fig. 2c shows, the distributions, numbers and types of *cis*-elements vary among the promoter sequences. Nevertheless, most of the promoters contain a number of MYB- and MYC-binding sites, except for the promoter of *CsATG12*, which lacks MYC-binding sites. In addition, most of the promoters of *CsARGs* contain ABA- and MeJA-responsive elements, and some of them contain GA, SA, auxin, cold, drought, defense and stress (TC-rich repeats)-responsive elements. In addition, all of the promoters of *CsARGs* contain numerous light-responsive elements, including G-boxes, MREs, Box-4, and AE-boxes (not shown in Fig. 2c). Overall, each *CsARG* may play a vital role in responding to circadian variation, hormones, and biotic and abiotic stresses.

### Protein-protein interaction networks of *Cs*ARGs

To investigate the interactions among *Cs*ARGs in tea plants, the ortholog groups of CsARGs, which originated from *Arabidopsis*, were used to construct PPINs. As a result, 35 orthologs of CsARGs were obtained from Inparanoid web server, and those 35 orthologs formed 360 protein-protein association patterns. The ARGs were determined to be closely related to one another, except for ATG14, which has not been fully studied in *Arabidopsis*. Among these proteins, 24 ARGs, including ATG9, ATG7, and ATG1c, were reported or predicted to interact with more than 20 ARGs, suggesting that the occurrence of autophagy requires interactions among numerous ARG proteins.

### Conserved domain and motif distribution analysis of *Cs*ATG8s

**Fig. 3:**
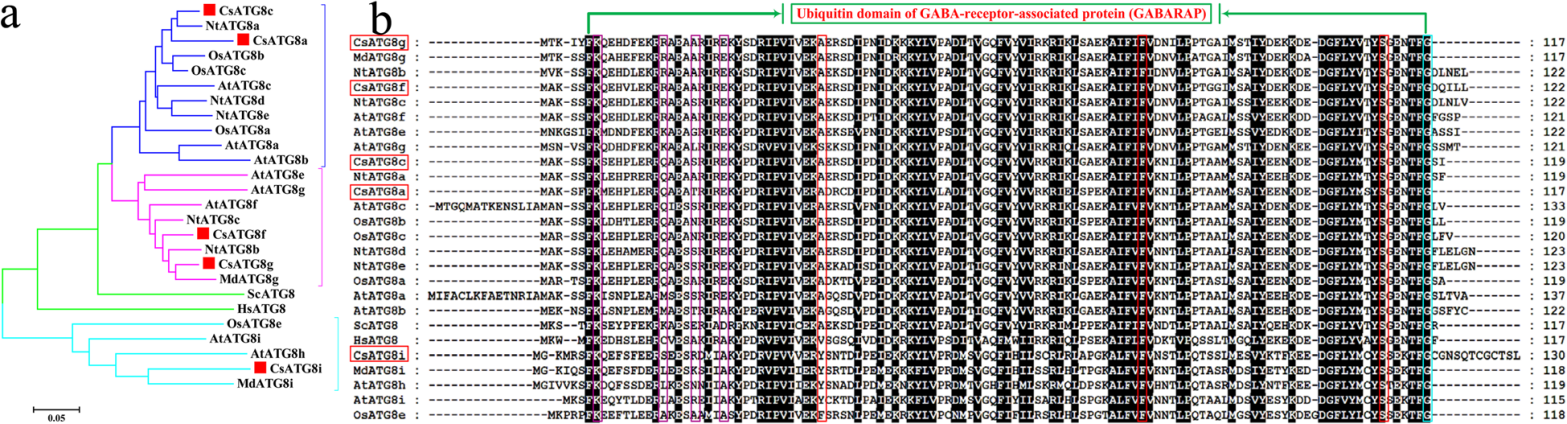
Conserved domains analysis of CsATG8s. **a** Phylogenetic analysis of CsATG8s and known ATG8s in *Arabidopsis*, *Oryza sativa*, *Malus domestica*, *Saccharomyces cerevisiae* and Humans. CsATG8s were highlighted with red boxes. **b** Amino acids alignment analysis of CsATG8s and known ATG8s in *Arabidopsis*, *Oryza sativa* and *Malus domestica*. Three putative ATG7 binding sites were contained in the red boxes respectively, four putative tubulin binding sites were contained in the pink boxes, and a conserved glycine (G) residue was framed in the bright blue box

As members of the UBQ superfamily, ATG8s coupled with their conjugation system are key components for autophagy. In our study, to clearly understand the regulatory mechanisms governing these proteins, the bioinformatic characteristics of *Cs*ATG8 subfamily proteins were further explored. As Fig. 3 shows, a total of 5 *Cs*ATG8s were identified in tea plants based on homologous alignment analysis. Phylogenetic analysis results showed that *Cs*ATG8s were subdivided into 3 clades. Among these proteins, *Cs*ATG8a/c and *Cs*ATG8g/f were clustered into one group, and *Cs*ATG8i was aligned closely with MdATG8i (Fig. 3). Motif distribution analysis results showed that *Cs*ATG8s contain motifs 1–4, and *Cs*ATG8i contains an additional motif 7 at the C-terminus (Additional file 2). All of these *Cs*ATG8s proteins contain conserved GABARAP domains, four putative tubulin binding sites, three ATG7 binding sites, and a conserved glycine (G) residue. In addition, we found that the conserved G residues in both *Cs*ATG8a and *Cs*ATG8g were directly exposed at the C-terminus (Fig. 3), suggesting that the functions of *Cs*ATG8a and *Cs*ATG8g involved in autophagy may be distinct from those of the other three proteins. These proteins may not require the cysteine protease Atg4 to cleave the C-terminus but may directly bind to the E1-like enzyme Atg7.

### Expression profiles of *CsARGs* in various tea plant tissues

**Fig. 4:**
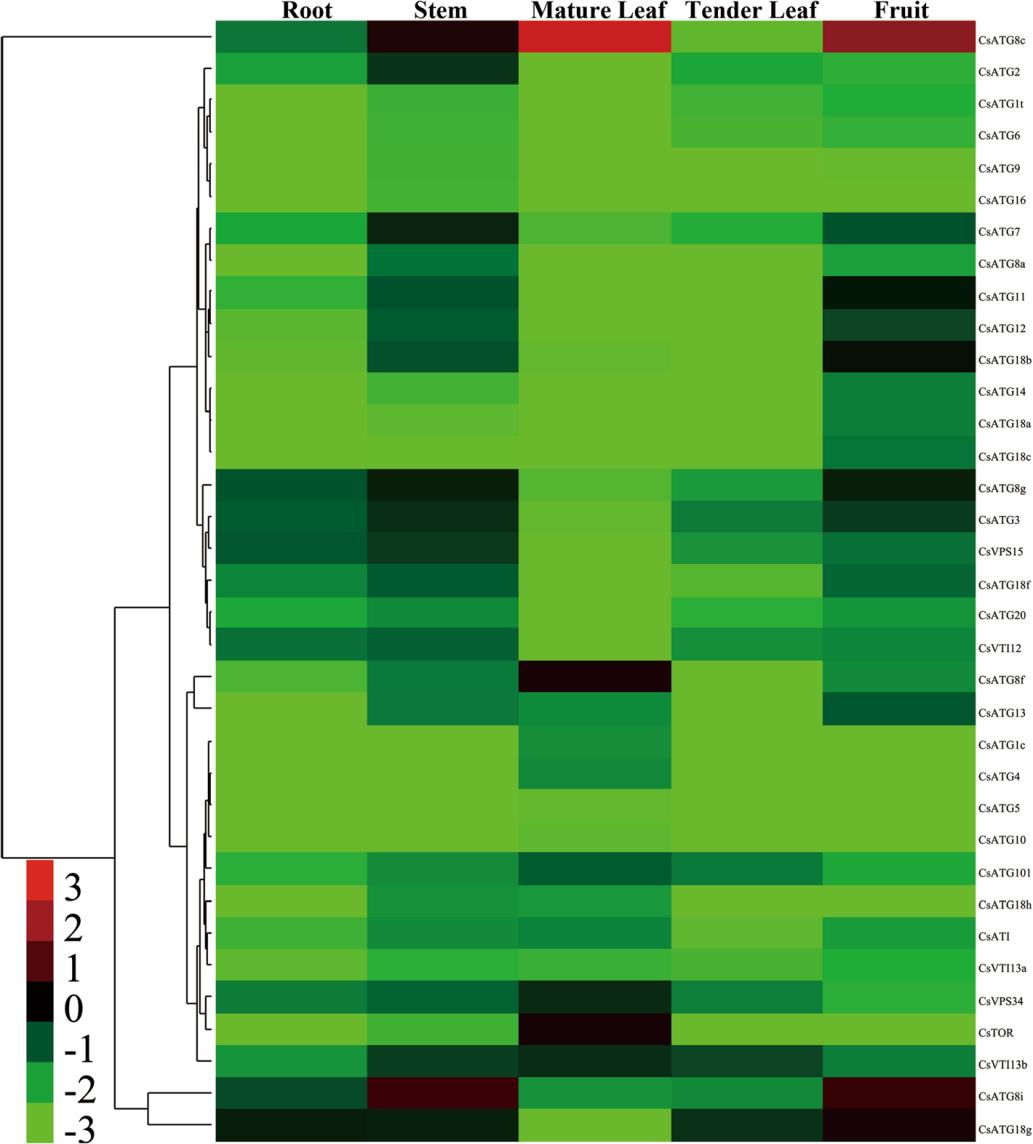
Expression profiles of *CsARGs* in different tea plant tissues. The transcription abundances of *CsARGs* in different tissues were monitored by using qRT-PCR, and the results were calculated by using 2^–ΔCt^ method. *CsPTB* was chose as actin gene. The heat map was generated by using Cluster 3.0 software. The colorbar was displayed on the lower-left of the heat map, red and green colors represent higher and lower expression levels respectively

To confirm the tissue specificity of *CsARGs*, the roots, stems, mature leaves, tender leaves, and seeds of tea plants were obtained for qRT-PCR analysis. The results showed that the transcription of all *CsARGs* was detected among the above mentioned tissues, although the mRNA level of each *CsARG* varied across the various tissues (Fig. 4). In addition, we found that most *CsARGs* exhibited higher transcription abundances in stems and seeds, suggesting that autophagy plays important roles in the development of stems and seeds in tea plants. Moreover, we found that the *CsATG3*/*7*/*101*, *Cs*V*PS15*/*34*, *CsATI*, *CsVTI12*/*13b*, *CsATG8s* and *CsATG18s* subfamily genes were highly expressed in different tissues. Notably, *CsATG8c* was significantly expressed in mature leaves and seeds, and *CsATG8i* was dramatically expressed in stems and seeds. In brief, our results found that the expression patterns of the *Cs*ARGs varied across different tissues, but some of them showed a degree of tissue specificity, suggesting that *CsARGs* mediated the growth and development of tea plants.

### Differential expression of *CsARGs* in response to hormone treatments

**Fig. 5:**
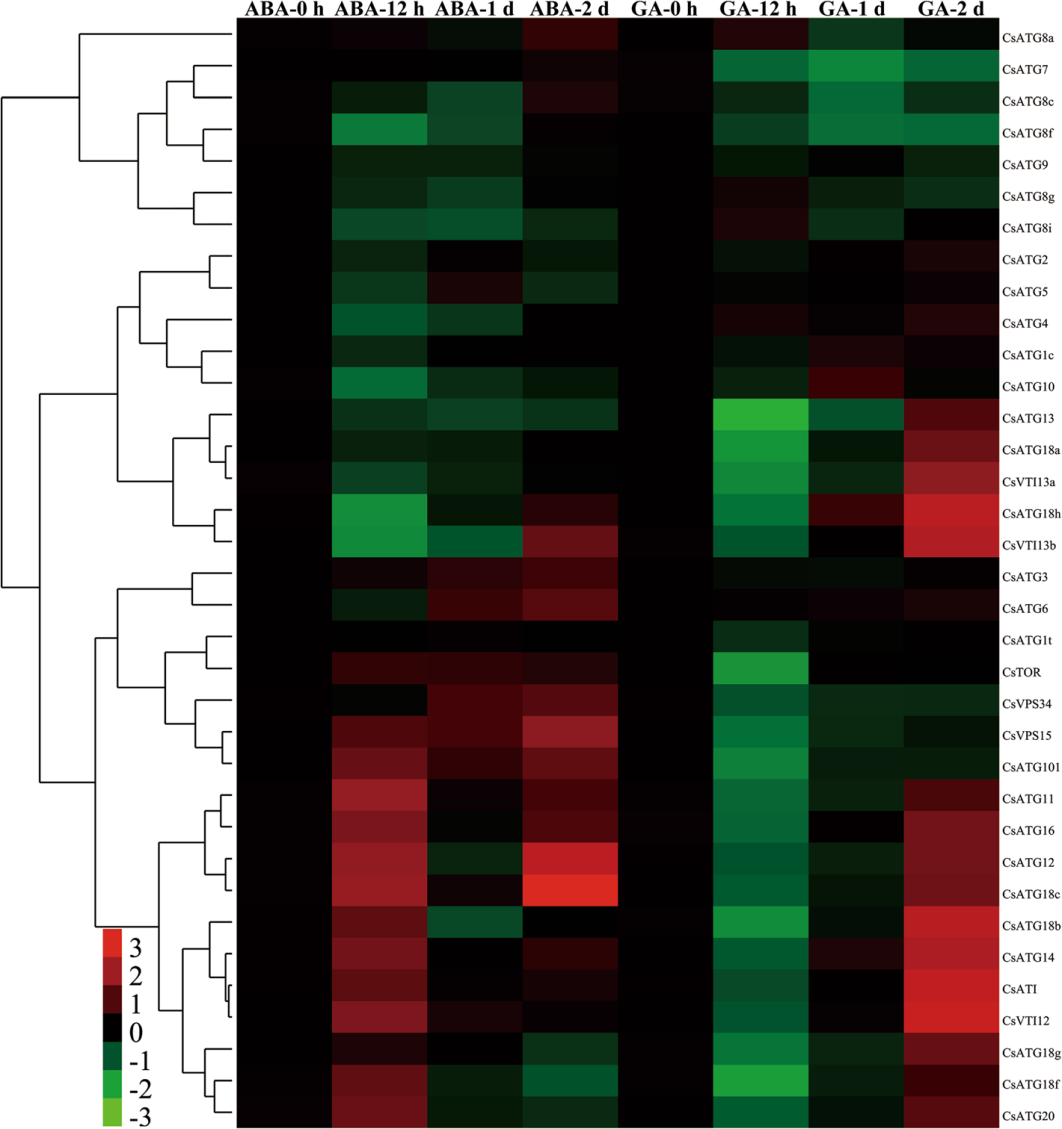
Temporal-spatial expression patterns of *CsARGs* in response to hormone treatments. The expressions of each *CsARG* gene within 2 d of ABA and GA treatments were performed by using qRT-PCR technique respectively. *CsPTB* was chose as the actin gene. The final results were calculated with 2^–ΔΔCt^ method, and the samples that collected at 0 h were set as control. The heat map was generated by using Cluster 3.0 software. The colorbar was displayed on the lower-left of the heat map, red and green colors represent higher and lower expression levels, respectively

To elucidate the comprehensive roles of *CsARGs* under ABA and GA treatment conditions, we analyzed the expression pattern of each *CsARG*. Under ABA treatment conditions, we observed that multiple *CsARGs* were highly induced after 12 h and/or 2 d of ABA treatment. Among these genes, the expression levels of *CsATG6*/*11*/*12*/*14*/*16*/*18b*/*18c*/*18f*/*20*/*101* and *CsVTI12*/*VTI13b* were induced more than 2-fold for at least at one time point. Meanwhile, some genes, such as *CsATG3*/*11*/*18c*/*101*/V*PS15*/*TOR*, were directly upregulated during the entire ABA treatment period (Fig. 5). In contrast, *CsARGs* exhibited contrary expression profiles under GA stress compared to ABA stress. Most *CsARGs* initially decreased but significantly increased after 2 d of GA treatment. Among these genes, the expression levels of 13 genes, including *CsATG12/14/16/18a/18b/18c/18 g/18 h/20/ATI/VTI12/VTI13a/VTI13b*, were increased more than 2-fold after 2 d of GA treatment. Furthermore, 6 genes, *CsATG7*/*8c*/*8f*/*101*/*VPS15*/*VPS34*, were negatively downregulated throughout GA stress periods. These results indicated that *Cs*ARGs are required for the response to hormone treatments in tea plants.

### Expression patterns of *CsARGs* in response to different abiotic stresses

**Fig. 6:**
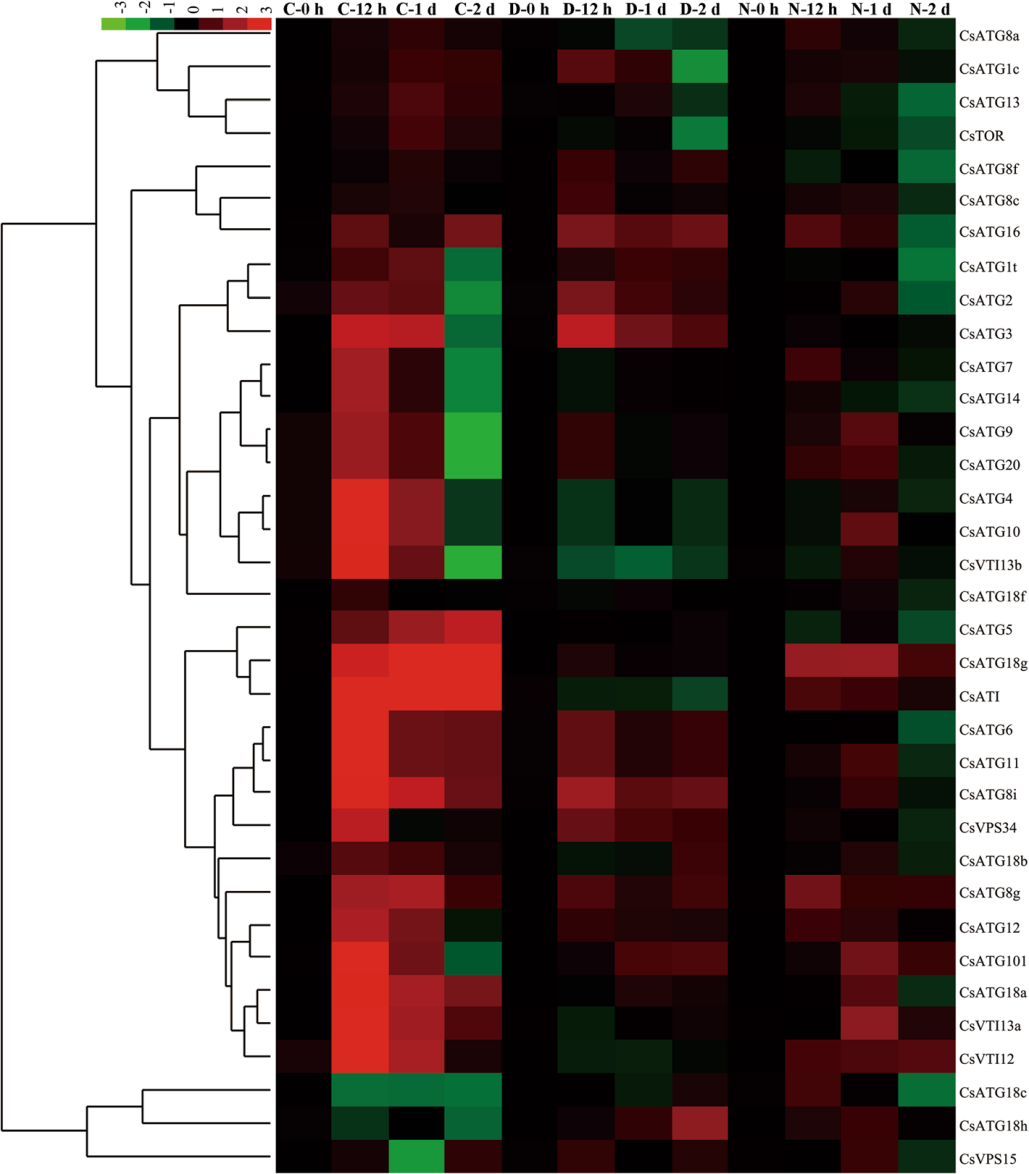
Temporal-spatial expression patterns of *CsARGs* in tea plant under various abiotic stresses. The expressions of each *CsARG* gene within 2 d of cold, drought, NaCl treatments were performed by using qRT-PCR technique respectively. *CsPTB* was chose as actin gene. The final results were calculated with 2^–ΔΔCt^ method, and the samples that collected at 0 h were set as control. The heat map was generated by using Cluster 3.0 software. The colorbar was displayed on the upper- left of the heat map, red and green colors represent higher and lower expression levels, respectively

Similarly, to explore the temporal expression patterns of *CsARGs* under abiotic stress conditions, the related expression level of each *CsARG* was determined by qRT-PCR. As shown in Fig. 6, the expression levels of all *CsARGs* were regulated to different degrees under various abiotic stress conditions.

During the CT period, the expression levels of almost all *CsARGs* were upregulated at different processing time points, except for 2 genes, *CsATG18c*/*h*, which were downregulated throughout the CT period. In addition, 20 induced *CsARGs* showed the highest expression levels after 12 h of CT. Specifically, the expression levels of *CsATG3*/*4*/*6*/*8i*/*10*/*11*/*18a*/*18 g*/*101*/*VTI12*/*VTI13a*/*VTI13b*/*VPS34* were more than 5-fold higher than those at 0 h of CT. In addition, *CsATG5*/*ATG18g*/*ATI* expression was gradually induced within 2 d of CT. Under DT conditions, 15 *CsARGs* were induced at different DT time points, and most of these genes showed the highest expression levels after 12 h of DT. The expression levels of *CsATG2*/*3*/*6*/*8i/11*/*16*/*VPS34* were more than 2-fold higher than those at 0 h of DT. Moreover, *CsATG18h* and *CsATG101* were gradually upregulated as the DT time extended. Conversely, the expression of the remaining *CsARGs*, such as *CsATG4*/*7*/*8a*/*10*/*14*, was slightly deduced or not affected by DT. Within NT periods, many *CsARGs* were also induced to different degrees. In contrast to CT, most of the induced *CsARGs* showed the highest expression levels after 1 d of NT but were deduced following the extended treatment time. In particular, the expression levels of *CsATG9/10/18a/18 g/101/VTI13* were increased more than 2-fold after 1 d of NT compared to the control. However, *CsATG8g*/*12*/*16*/*ATI* was significantly upregulated after 12 h of NT. Taken together, these results demonstrated that *CsARGs* play central roles in responding to abiotic stress in tea plants.

### Differential expression of *CsARGs* in different tea plant cultivars during CA periods

**Fig. 7:**
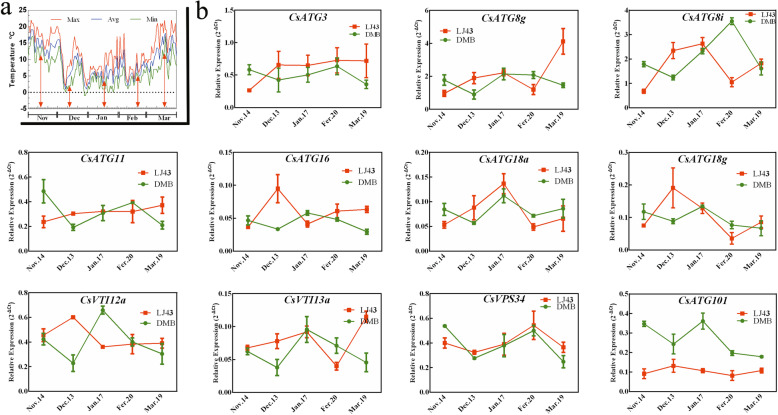
Expression analysis of *CsARGs* during CA periods in 2018–2019. **a** Changes in air temperature from November 2018 to March 2019. The maximum (Max), average (Avg) and minimum (Min) daily temperatures were respectively indicated by red, blue and green colored lines. The red arrows represent sampling days. **b** The relative expression levels of 11 *CsARGs* in two tea cultivars during CA periods in 2018–2019. The expression profiles of *CsARGs* in two tea cultivars were displayed with different colored lines. The results were calculated by using the 2^–ΔCt^ method with *CsPTB* as the actin gene. Data are shown as the means ± SE (*n* = 3)

The differential expression patterns of *CsARGs* were compared in different tea plant cultivars (the cold-resistant cultivar ‘Longjing43’ and the cold-susceptible cultivar ‘DaMianBai’) within CA periods. Eleven *CsARGs*, which were confirmed to be notably induced under CT conditions, as shown in Fig. 7, were selected for analysis by qRT-PCR. The results showed that these 11 genes presented different expression patterns in ‘Longjing43’ and ‘DaMianBai’ during the CA period in 2018–2019. Specifically, these 11 genes showed contrary expression patterns from November 14th to December 13th in ‘Longjing43’ and ‘DaMianBai’. With the exception of *CsVPS34*, the other *CsARGs* were continuously upregulated in ‘Longjing43’, but all of them were gradually reduced in ‘DaMianBai’ from November 14th to December 13th. In contrast, the transcription levels of these 11 genes were all increased in ‘DaMianBai’ from Dec 13th to Jan 17th, but many *CsARGs*, such as *CsATG16*/*18 g*/*101*/*VTI12,* were decreased in ‘Longjing43’. Notably, we observed that the transcription level of *CsATG101* was lower in ‘LongJing43’ than in ‘DaMianBai’ throughout the CA period. These results indicated that these *CsARGs* play important roles in the response of tea plants to cold resistance, but their regulatory mechanisms may vary among different cultivars.

## Discussion

### *CsARG*s are involved in different stages of autophagy in tea plants

Autophagy is a catabolic degradation pathway that is essential for degrading long-lived proteins, protein aggregates, and damaged organelles [55]. It has been proven that autophagy is highly conserved from yeast to humans and is the result of the interactions of many proteins. To date, more than 30 ATG-related genes have been identified in many eukaryotes, and those *ATGs* encode many core proteins involved in the entire process of autophagy, extending from induction to the degradation, recovery and recycling of autophagosomes. As the tea plant is a type of evergreen wooden plant, the recycling of some broken or discarded macromolecular substances plays an important role in the special development period or in the resistance to stresses in this plant. In the present study, a total of 35 *CsARG*s were identified in the tea plant genome. Some *CsARG*s, such as *CsATG8* and *CsATG18*, exhibit multiple copies in the tea plant genome, and similar results were also observed in many other species, such as *Oryza sativa* [17], *Nicotiana tabacum* [18], *Vitis vinifera* [20], *Musa acuminate* [21], and *Setaria italic* [22]. In addition, the results of phylogenetic and protein domain analysis further confirmed that ATGs are highly homologous among different plant species. In yeast, ATG proteins are divided into four functional groups based on their roles in the autophagy process [56]. Similarly, the identified *Cs*ARGs also constitute a relatively complete autophagic machinery in which they function in forming the ATG1 kinase complex (*Cs*ATG1s/13, *Cs*TOR), Class III PI3K complex (*Cs*ATG6/14, *Cs*VPS15/34), ATG9 recycling complex (*Cs*ATG2/9/18 s), Atg8-lipidation system (*Cs*ATG3/4/7/8 s) and Atg12-conjugation system (*Cs*ATG5/7/10/12/16).

To date, there have been numerous reports on the functional analysis of ATG14 in mammals but few in plants. In mammals, the human ATG14 homolog, hAtg14/Barkor/Atg14L, has been shown to be the sole specific subunit in the phosphatidylinositol 3-kinase (PI3-kinase) complex. Atg14L was observed to interact with Beclin-1/2 through their coiled-coil domains and proved to be the targeting factor for PI3KC3 to the autophagosome membrane [57, 58]. Similarly, ATG14 is only integrated into phosphatidylinositol 3-kinase complex I to direct the association of complex I with the pre-autophagosomal structure (PAS) in yeast [59]. In this study, we identified an ATG14 homologous protein, referred to as *Cs*ATG14, in tea plants. Bioinformatic analysis results predicted that *Cs*ATG14 is a hydrophilic protein and contains a coiled-coil motif at the N-terminus region from 10 to 367 aa, suggesting that *Cs*ATG14 may interact with Beclin-1 (designated *Cs*ATG6 in our study) to serve as a scaffold for recruiting class III phosphatidylinositol-3-kinase (PIK3C3). However, this possibility requires further verification by using the yeast double hybrid (Y2H) method.

As a highly conserved ubiquitin-like protein, ATG8 is activated by conjugation to lipid phosphatidylethanolamine (PE) to form the ATG8-PE adduct, thereby participating in autophagosome formation and phagophore expansion [60, 61]. During conjugation, the C-terminus of ATG8 must be cleaved by a cysteine protease, Atg4, to expose a glycine residue [62, 63]. In this study, five ATG8 isoforms were identified, and they displayed high sequence similarity to AtATG8s, NtATG8s and OsATG8s. However, a conserved glycine residue for lipidation was directly exposed at the C-terminus of *Cs*ATG8a/g, and similar phenomena were also observed in MdATG8g/i, AtATG8h/i and OsATG8e, suggesting that ATG4 may not be necessary for the conjugation of ATG8-PE. In addition, ATG8 can also interact with its specific substrates or receptors via an Atg8 interacting motif (AIM) in the target proteins during selective autophagy. Recently, two plant-specific proteins in *Arabidopsis*, termed ATI1 and ATI2, were identified and proven to interact with AtATG8f and AtATG8h [64]. In our study, however, only one unique ATI homologous gene, *Cs*ATI, was identified. Sequence alignment analysis found that *Cs*ATI also contains two putative AIMs (17–20, 267–270) and a predicted transmembrane domain (242–259) (Additional file 2), suggesting that *Cs*ATI can also bind one of five *Cs*ATG8 isoforms in tea plants. To clarify whether *Cs*ATI interacts with *Cs*ATG8 isoforms, Y2H could be performed by using a *Cs*ATG8 isoform recombinant plasmid as bait and a *Cs*ATI recombinant plasmid as prey.

During macroautophagy, a v-SNARE complex, including v-SNARE VTI1, Rab-like GTP-binding protein (YKT6), and syntaxin (VAM3), contributes to the maturation and fusion of autophagosomes to lysosomes/vacuoles [65]. In plants, three VTI1-type SNARE members (VTI11, VTI12, VTI13) have been identified [66]. Sanmartin et al. (2007) suggested that VTI12 and VTI11 might be involved in trafficking to storage and lytic vacuoles in vegetative and seed tissues in *Arabidopsis*, respectively [67]. Moreover, VTI13 participates in the trafficking of cargo to the vacuole within root hairs and plays an essential role in the maintenance of cell wall organization in *Arabidopsis* [68]. In our study, *Cs*VTI12 and *Cs*VTI13a/b were all predicted to contain typical v-SNARE domains (Fig. 2b), and phylogenetic analysis showed a close relationship with other VTI1s (Fig. 1), suggesting that *Cs*VTI1s are essential for autophagy and may mediate various protein transport pathways.

### *CsARG*s mediate the growth and development of tea plants

Under normal growth conditions, autophagy serves as a housekeeping process to degrade unwanted proteins, organelles and damaged cytoplasmic contents. Recently, numerous studies observed that autophagy mediates plant senescence [69]. Indeed, autophagy is also necessary for anther and seed development [26, 30, 70], root elongation [71], and chloroplast recycling [72]. In the present study, we found that the transcription abundances of most *CsARGs* were higher in stems and seeds than in other tissues, which indicates that autophagy may mediate nutrient allocation or recycling from source tissues to sink tissues in tea plants. Specifically, *CsATG8s* subfamily genes showed high transcription levels in all detected tissues, which demonstrates that they play a notable role in modulating tea plant growth and development. A similar result was also observed in *Arabidopsis*, where *AtATG8s* were distinctly expressed throughout the plant [73]. As core ATG proteins, ATG8s have been used as very convenient markers to monitor autophagic activity and play vital roles in regulating nitrogen remobilization efficiency and grain quality in plants [74]. For instance, overexpression of *OsATG8b* increased the nitrogen recycling efficiency to grains in transgenic plants while reducing nitrogen recycling efficiency and grain quality in *OsATG8b*-RNAi transgenic plants [75]. Similarly, *ATG8a*, *ATG8e*, *ATG8f* and *ATG8g* overexpressed in *Arabidopsis* could promote autophagic activity and improve nitrogen remobilization efficiency and grain filling in transgenic plants [76]. In our study, both *CsATG8c* and *CsATG8i* were strongly expressed in tea seeds, suggesting that the high mRNA levels of these two genes may promote nitrogen remobilization efficiency in tea seeds. From this perspective, selecting tea plant germplasms with higher *CsATG8s* transcription abundances may be employed to improve tea seed quality and thus guarantee the seedling emergence rate and survival rate.

Chloroplasts are specific energy converters of higher plants and photoautotrophs, and nearly 80% of the total leaf nitrogen is stored in chloroplasts in C_3_ plants [77]. More recent findings determined that chloroplasts could be degraded by autophagy to serve as a principal nitrogen source for recycling and remobilization during leaf senescence. Indeed, it has been reported that autophagy also contributes to leaf starch degradation [78]. Tea plants are evergreen and C_3_ plants, and the numbers of chloroplasts in leaves gradually increase from tender leaves to mature leaves and subsequently decrease with leaf senescence. In the present study, we analyzed *CsARG* expression in both mature and tender leaves, and the results showed that 11 *CsARGs* exhibited more than 3-fold higher expression levels in mature leaves than in tender leaves, indicating that autophagic activity is changed following the maturation of leaves. The higher autophagic activity in mature leaves may be attributed to prolonged leaf longevity, which maintains the evergreen nature of tea plants for an extended period of time. However, to illustrate this assumption, the expression of *CsARGs* in various tissues at different growth stages in different tea plant cultivars should be determined.

### *CsARGs* improved abiotic stress tolerance in tea plant

In addition to mediate plant growth and development, autophagy also plays a critical role in plant resistance to various stresses, such as nutrient deficiency, oxidation stress, cold, drought, salt, wounding, heavy metal, and pathogen attack. Currently, overexpression and mutation methods have been widely employed to explore the functions of *ATG* genes in different species. For example, overexpression of an ATG gene, *MdATG18a*, in apple plants could enhance drought resistance, probably by inducing greater autophagosome production and higher autophagic activity [79]. In addition, overexpression of *MdATG18a* also regulated the expression of many genes involved in anthocyanin biosynthesis, sugar metabolism, and nitrate uptake and assimilation and finally promoted soluble sugar and anthocyanin accumulation, starch degradation and nitrate utilization improvement in response to N depletion [80]. Aside from *ATG18a*, *ATG3*/*5*/*7*/*8 s*/*10* has also been reported to play critical roles in addressing different stresses. Overexpression of *MdATG3s* enhanced tolerance to multiple abiotic stresses in both transgenic *Arabidopsis* and apple plants [81]. Overexpression of *AtATG5* or *AtATG7* in transgenic *Arabidopsis* activated AtAtg8-PE conjugation, autophagosome formation, and autophagic flux, thereby increasing the tolerance of necrotrophic pathogens and oxidative stress, retarding aging and improving growth and seed yields [34]. Indeed, MdATG8i was proven to interact with MdATG7a and MdATG7b, and overexpression of *MdATG8i* enhanced tolerance to nutrient starvation in both transgenic *Arabidopsis* and apple plants [22]. In the present study, the expression of *CsATG3/5/7/8 g/8i/18a/18 g* was quickly induced by cold, drought, and NaCl treatments. Furthermore, the promoters of these genes contain a series of *cis*-acting elements that may be involved in the response to environmental stresses or hormones, indicating that autophagy is induced in tea plants under adverse environmental conditions, and many *CsATG* genes participate in addressing different stresses.

Autophagy is also closely related to sugar signaling. The central energy-sensing SnRK1 acts as a positive regulator, which acts upstream of TOR on sugar-phosphate perception to activate autophagy, and TOR kinase acts as a negative factor to inhibit autophagy [82]. Accumulating evidence has proven that many genes involved in sugar metabolism, transport and signaling are differentially expressed in tea plants during abiotic stress conditions; for example, *CsSnRK1.2* expression is induced, *CsSnRK1.*1 expression is not affected, and *CsSnRK1.3* expression is notably reduced during CA periods in tea plants [83, 84]. Combined with our results, the expression profile of *CsTOR* did not show a strictly contrary tendency to *CsSnRK1*, whereas we found that the expression of *CsTOR* was slightly induced under cold conditions but was reduced under drought and NaCl conditions, suggesting that autophagy could be activated by TOR-independent pathways and that SnRK1 could also mediate autophagy through a TOR-independent mechanism in tea plants under certain stress conditions. Autophagy is also regulated by phytohormones. Under normal conditions, TOR kinase phosphorylates PYL receptors and represses ABA signaling, whereas ABA signaling represses TOR kinase activity through the phosphorylation of Raptor B mediated by SnRK2 under stress conditions [85]. In the present study, however, we found that the expression of *CsTOR* was slightly induced under ABA treatment conditions, which indicates that the inhibition of TOR is not simply affected by the transcription level but is primarily influenced at the posttranslational level during stress conditions. At present, few studies have explored the relationship between autophagy and GA. Kurusu et al. (2017) found that *OsATG7* mutated in rice could reduce the endogenous level of active forms of gibberellins (GAs) in anthers of an autophagy-defective mutant, Osatg7–1, during the flowering stage, which suggested that autophagy mediated the biosynthesis of GAs in rice [86]. In the present study, we observed that two-thirds of *CsARGs* were induced after 2 d of GA treatment, which indicates that there is a close relationship between GA metabolism and autophagy, but the specific regulatory mechanism governing this phenomenon warrants further investigation.

Cold acclimation (CA) is an indispensable process to increase the cold tolerance of tea plants. During CA, higher ROS contents and lower SOD activity were observed in the cold-susceptible cultivar ‘DaMianBai’ than in the cold-resistant cultivar ‘Longjing43’ [87]. In addition, many genes related to ROS production and scavenging were induced and deduced in ‘DaMianBai’ under CA conditions, demonstrating that the stimulation of ROS-scavenging genes was a principal strategy for tea plants in response to cold stress. However, in addition to ROS-scavenging genes, autophagy could also inhibit ROS production under stress conditions [27]. In our study, we found that the expression of 10 *CsATGs* was higher in ‘Longjing43’ than in ‘DaMianBai’ during CA periods (from Dec. 13 to Jan. 17), which indicates that autophagic activity may be higher in the cold-resistant cultivar than in the cold-susceptible cultivar during CA periods. However, we found that another ATG gene, *CsATG101*, was more highly expressed in ‘DaMianBai’ than in ‘Longjing43’ throughout the CA period. At present, ATG101 is well-known as a component of the ULK1 complex, which serves as a stabilizer of ATG13 in cells. In mammals, ATG101 is required for maintaining tissue homeostasis in both adult brains and midguts, but the physiological role of ATG101 has not been fully elucidated in plants. Therefore, the specific regulatory mechanism governing the response of *CsATG101* to CA needs to be further studied. In summary, based on the differential expression patterns in different cultivars, we believe that these 11 *CsATG* genes may serve as putative molecular markers for the breeding of cold-resistant tea plants in future research. However, to confirm the abovementioned claims, functional analyses of these *CsARG* genes, including autophagosome detection, subcellular analysis, promoter cloning and analysis, protein interactions and yeast complementation, and overexpression analysis in *Arabidopsis* or *Nicotiana tabacum,* should be performed in the future.

## Conclusions

In the present study, a total of 35 *CsARGs* were identified, and each *Cs*ARG showed a close relationship to its homologue from other plant species. The transcriptional abundances of *CsARGs* vary in different tissues, but some of them show a certain degree of tissue specificity. Under various abiotic stress conditions, most *CsARGs* were induced at different treatment time points, which indicated that *CsARGs* play central roles in the response to abiotic stress in tea plants. In addition, 10 *CsARGs* were more highly expressed in the cold-resistant cultivar than in the cold-susceptible cultivar during CA periods; however, *CsATG101* showed the opposite tendency, suggesting that these genes serve as putative molecular markers for cold-resistant breeding of tea plant. In this study, we performed a comprehensive bioinformatic and physiological analysis of *Cs*ARGs in tea plants, and these results may pave the way for further research on the molecular mechanism governing autophagy in tea plant growth, development and response to stress.

## Methods

### Plant materials and stress treatments

Three-year-old clonal potted seedlings of the ‘LongJing43’ cultivar, which were planted in the greenhouse of the Tea Research Institute of the Chinese Academy of Agricultural Sciences (TRI, CAAS, N30°10′, E120°5′), were used for tissue-specific analysis. Tissues including roots, stems, mature leaves, tender leaves, and seeds were sampled and quickly frozen in liquid nitrogen to store at − 80 °C. Three independent biological replicates were performed, and each replicate contained three seedlings with similar growth states.

One-year-old clonal potted seedlings of the ‘LongJing43’ cultivar, which were cultivated at the experimental base of Qingdao Agricultural University (QAU, N36°33′, E120°4′), were employed for cold, drought, salt, and hormones treatments. Before treatment, all seedlings were cultured in a growth chamber under the following growth conditions: temperature, 23 ± 0.5 °C; lighting time, 14 h/10 h (light/dark); and humidity, 75%. Cuttings with the same growth potential were used to process different treatments as described by Qian et al. (2016) [46] with some modifications. For cold treatment (CT), the temperature of the growth chamber was plummeted to 4 °C without changing any other growth conditions. PEG-6000 (10% (w/v)) and 250 mmol·L^− 1^ NaCl were used to imitate drought (DT) and salt treatment, respectively. To proceed hormone treatments, 100 μmol·L^− 1^ ABA and 100 μmol·L^− 1^ GA were sprayed onto the surfaces of tea leaves. During stress treatment periods, the other aspects of the growth conditions were maintained the same as the control. All of these treatments were carried out for 2 d, with samples of the third and/or fourth mature leaves from the terminal bud being taken at 0, 12, 24 and 48 h after treatment. For each stress treatment, the samples collected at the 0 h time point were taken as controls. All samples were quickly frozen in liquid nitrogen and stored at − 80 °C. Each stress treatment contained three biological replicates.

A cold-resistant cultivar, ‘LongJing43’, and a cold-susceptible cultivar, ‘DaMianBai’, as reported by Wang et al. (2019) [87], were used for natural cold acclimation (CA) analysis in 2018–2019. Both tea cultivars were 18 years old and cultivated at the Tea Research Institute of the Chinese Academy of Agricultural Sciences (TRI, CAAS, N30°10′, E120°5′). The sampling methods were performed as described by Qian et al. (2018) [49].

### Identification of *CsARG*s in tea plant

To identify putative *CsARG*s in tea plant, four methods were used to search the homologous sequences of *ARGs* in published tea plant genomes and transcriptomes. First, ‘autophagy’ or ‘ATG’ as a keyword was searched in the Tea Plant Information Archive database (TPIA, http://tpia.teaplant.org/index.html) [88]. Second, ‘autophagy’ or ‘ATG’ as a keyword was searched in published transcriptome data [83]. Third, the nucleotide and protein sequences of ATG-related genes in *Arabidopsis* (44), rice (33), banana (31), grapevine (35), and tobacco (29) were retrieved from the Phytozome v12.1 database (JGI, https://phytozome.jgi.doe.gov/pz/portal.html), and then they were all used as references to perform BLASTn and BLASTx against the TPIA database. Fourth, the raw Hidden Markov Models (HMM) of ATG-related protein domains were also used for predicting and identifying ATG-related proteins from tea plant. Briefly, the ATG protein sequences of *Arabidopsis* were regarded as references to query the matched HMM from the Pfam database (http://pfam.xfam.org/), and then we used the HMMER 3.0 software to search the homologous sequences from the tea plant protein database through entering “hmmsearch --domtblout output_result.txt --cut_tc Pfam.hmm all.pep.fasta” codes into cmd.exe. After removing redundant sequences, all of the retained protein sequences were searched in the NCBI conserved domain database (https://www.ncbi.nlm.nih.gov/Structure/bwrpsb/bwrpsb.cgi) [89] to verify the presence of ATG-related domains and also performed BLASTp analysis for further guaranteeing whether they are belongs to ATG-related proteins. In addition, partial proteins that have been confirmed to interact with ATGs were also identified and analyzed using the same methods in this study.

### Bioinformatics analysis of CsARGs in tea plant

The open reading frame (ORF) and potential amino acids were searched using ORF finder web (https://www.ncbi.nlm.nih.gov/orffinder/). Molecular weights and theoretical pI were calculated using the ProtParam tool (http://web.expasy.org/protparam/). Signal peptides and transmembrane regions were predicted using The SignalP 4.1 Server (http://www.cbs.dtu.dk/services/SignalP/) and the TMHMM Server v.2.0 (http://www.cbs.dtu.dk/services/TMHMM/), respectively. Plant-mPLoc (http://www.csbio.sjtu.edu.cn/bioinf/plant-multi/), WoLF PSORT (http://wolfpsort.org/), TargetP 1.1 Server (http://www.cbs.dtu.dk/services/TargetP/), MitoProt (https://ihg.gsf.de/ihg/mitoprot.html), and YLoc (http://abi.inf.unituebingen.de/Services/YLoc/webloc.cgi) were used to predict the subcellular locations of CsARGs.

### Phylogenetic analysis

To explore the evolutionary relationships among the ARG proteins in various plant species, an unrooted phylogenetic tree of the ARGs identified in *Arabidopsis*, rice, tobacco, and foxtail millet was constructed. In brief, the amino acid sequences of *At*ARGs, *Os*ARGs, *Nt*ARGs, and *Si*ARGs were downloaded from Phytozome v12.1 database. Next, these ARG proteins were aligned together with *Cs*ARGs to proceed to complement alignment analysis by using ClustX2.1. Subsequently, the alignment results were imported into MEGA 5.0 and converted into meg. format. After that step, the phylogenetic tree was generated by using MEGA 5.0 with the following parameters: the neighbor-joining method, 1000 repeated bootstrap tests, p-distance, and pairwise deletion. A circle tree was formed with a scale length of 0.1 and a cutoff value for consensus tree of 50% and was finally managed using the ITOL website (https://itol.embl.de/).

### Gene structure, protein domain distribution and *cis*-acting element analysis

The exon-intron structures of *CsARGs* were visualized by the GSDS 2.0 website (http://gsds.cbi.pku.edu.cn/) by comparing the coding sequences (CDSs) with their corresponding genomic sequences. Protein domains of *Cs*ARGs were determined using SMART online tools (http://smart.embl-heidelberg.de/). The 2000-bp upstream noncoding region sequences of each gene were used to predict *cis*-acting elements involved in responding to stresses and hormones by using PlantCARE software (http://bioinformatics.psb.ugent.be/webtools/plantcare/html/). To obtain the promoter sequences, the genome sequences of each *Cs*ARG gene were downloaded from the two published tea plant genomes (‘ShuChaZao’ and ‘YunKang10’) in the TPIA database. Then, a 2000-bp noncoding region sequence upstream of the translation initiation site (ATG) in each *Cs*ARG genome sequence was regarded as a promoter sequence for analyzing *cis*-acting element sites.

### Conserved domains and motifs analysis

Multiple amino acids of ATG8s and ATI originating from different species were used to align conserved domains according to Clustlx2.0 software, and the results were exported using Genedoc software. The conserved domains of *Cs*ATG8s were identified using the MEME website (http://memesuite.org/) with optimum motifs ≥5 bp and ≤ 50 bp and a maximum number of motifs 15.

### Construction of protein interaction networks

To construct the protein-protein interaction networks (PPINs) of *Cs*ARGs, the ortholog groups of *Cs*ARGs must be identified firstly from* Arabidopsis* due to there is no PPIs information of tea plant in the current PPI database. Here, the Blast search column in Inparanoid (http://inparanoid.sbc.su.se/cgi-bin/index.cgi) is used to determine the ortholog groups of *Cs*ARGs, and we only picked the orthologs originated from Arabidopsis to construct PPINs. Then, STRING (https://string-db.org, Ver10.5) was used to construct the PPINs of the ortholog groups. In brief, all geneID of obtained orthologs were uploaded into the ‘Multiple Proteins by Names/Identifiers’ column, and *Arabidopsis thaliana* was chosen as a reference for blast searching. Then, the matched STRING proteins were chosen to build PPINs. Finally, the result was uploaded into Cystoscape v3.7.2 to draw the interaction map.

### qRT-PCR analysis

Total RNA isolation and first-strand cDNA synthesis of all samples were performed as described by Qian et al. (2018) [49]. For qRT-PCR analysis, 20 μL reaction volumes, including 10 μL SYBR Premix Ex Taq, 1.6 μL forward/reverse primers, 2 μL cDNA and 6.4 μL distilled water, were analyzed on a Roche 384 real-time PCR machine (Roche). The qRT-PCR program began with 95 °C for 10 min, followed by 45 cycles of 94 °C for 10 s, 60 °C for 15 s and 72 °C for 12 s; thus, a melting curve was added. *CsPTB* [90], as an actin gene, was used to quantify the relative expression levels of each *CsARG* according to the 2^-ΔCt^ or 2^−ΔΔCt^ methods [91]. Three replications were generated for each RNA sample for quantitative analysis, and the qRT-PCR primers are listed in Additional file 1.

### Statistical analysis

The representative data of each *Cs*ARG are presented as the mean values ± standard error (± SE). In addition, heat maps were generated by Cluster 3.0 software, and the figures were plotted by GraphPad Prism 6.0 (GraphPad Software, Inc., LA Jolla, CA, USA).

## Supplementary Information

**Additional file 1: Table S1.** Primers information used in qRT-PCR detection.

**Additional file 2: Figure S1.** Motif distribution in CsATG8s subfamily members of the tea plant, *Arabidorpsis*, *Oryza sativa*, *Malus domestica*, *Saccharomyces cerevisiae* and Humans. Different motifs are represented by various colors. **Figure S2.** Alignment analyses of CsATI with AtATIs and OsATI. The TM-HMM-predicted transmembrane helix of CsATI (242–259) are highlighted with a yellow box. The putative N-terminal and C-terminal AIMs are highlighted with red boxes.

## Data Availability

The datasets generated and/or analyzed during the current study are available in this article and the additional files. The nucleotide and protein sequences of ATG-related genes in *Arabidopsis*, rice, banana, grapevine, tobacco, and foxtail millet are available in Phytozome v12.1 database (JGI, https://phytozome.jgi.doe.gov/pz/portal.html).

## Abbreviations

ATG: Autophagy
ARG: Autophagy-related gene
CA: Cold acclimation
CT: Cold treatment
DT: Drought treatment
ORF: Open reading frame
PI3K: Phosphoinositide 3-kinase
qRT-PCR: Quantitative real-time PCR
TOR: Target of rapamycin kinase
