# Supplementary material for: Genome-wide identification, characterization, and expression analysis of tea plant autophagy-related genes (CsARGs) demonstrates that they play diverse roles during development and under abiotic stress
Source: BMC Genomics. 2021 Feb 17;22:121. doi: 10.1186/s12864-021-07419-2 (PMC7891152; doi:10.1186/s12864-021-07419-2)
Supplement: Supplementary file 1 — Additional file 1: Table S1. Primers information used in qRT-PCR detection. [file 12864_2021_7419_MOESM1_ESM.docx]

**Table S1** Primer information used in qRT-PCR detection.

| **ID** | **Gene name** | **Forward/Reverse** | **Primer sequence (5' to 3')** |
| --- | --- | --- | --- |
| [XP_028071137.1](https://www.ncbi.nlm.nih.gov/protein/XP_028071137.1?report=genbank&log$=protalign&blast_rank=1&RID=H8C3FAEB014) | CsATG1c | Forward | CTCTGGAGTGTTGGTGCCATTCTG |
|  |  | Reverse | GTCAACCGCTCCACTGGATTACG |
| XM_028254805.1 | CsATG1t | Forward | CGGTGGCGCTGAAGCAAGTC |
|  |  | Reverse | AGGGTGATGGACGGAGGACAAG |
| XM_028214951.1 | CsATG2 | Forward | CCATCAATGAGGAGGCACTGCTTC |
|  |  | Reverse | GCTAGGTCAACACCACAGGGAATG |
| XM_028269562.1 | CsATG3 | Forward | AATCTCGTCTCCAAATGCCCTAC |
|  |  | Reverse | CAGCAGCTTCATATTCTTCTTCCAC |
| XM_028205167.1 | CsATG4 | Forward | CCACATGAAGTTCAGCCGGTAGTTG |
|  |  | Reverse | GGCGGATTGCGGGTATGAGTAA |
| XM_028239072.1 | CsATG5 | Forward | GAGGACGGAAGCACAGAAGTACG |
|  |  | Reverse | CTAAGATCAGAGCGGGTGGAGGAG |
| XM_028209545.1 | CsATG6 | Forward | AAGCAAAGAAATCAAGTGTCGGGAGT |
|  |  | Reverse | CCAGAATTGTTTGGCTGCATAGGAG |
| XM_028207488.1 | CsATG7 | Forward | CGGGGCTTGCTCCTATTGCTTC |
|  |  | Reverse | ATGCCAAGAGGTTGCTCAGTGC |
| XM_028204481.1 | CsATG8a | Forward | AGGCAGGCAGAAGCTACTCGTATAA |
|  |  | Reverse | CAACAGTCAGATCAGCAAGAACCAAG |
| XM_028257959.1 | CsATG8c | Forward | CTCGAAAGGAGGCAGGCAGAAG |
|  |  | Reverse | GGCAGCAGTAGGTGGCAAAATG |
| XM_028237334.1 | CsATG8f | Forward | TGCCGAGGCTGCCAGGATTA |
|  |  | Reverse | CCCCTGTTGGTGGCAAGACATT |
| XM_028213593.1 | CsATG8g | Forward | CCTGTGATAGTGGAGAAGGCTGAG |
|  |  | Reverse | CCTGTAGGCGGAAGGATGTTGT |
| XM_028202806.1 | CsATG8i | Forward | GATCGAGTTCCCGTGGTGGTTG |
|  |  | Reverse | TCCCAGGAGCCAGACGAAGC |
| XM_028219288.1 | CsATG9 | Forward | TTTCTGGAGGCTTTGCTGCTATTC |
|  |  | Reverse | GGACTACCAGAGACATCGCACCTT |
| XM_028214294.1 | CsATG10 | Forward | AAAAGGATCTCCCCGCAAACTC |
|  |  | Reverse | GACGAACCACCTGCCCAACGAC |
| XM_028237709.1 | CsATG11 | Forward | GGGTGGCTGGATTTGCTGACT |
|  |  | Reverse | CACGGTGCCTTGACAATACGC |
| XM_028206145.1 | CsATG12 | Forward | GTCAACAGTGCCTTCTCACCCAA |
|  |  | Reverse | CACCAGCTTACCATCGAAACCAAA |
| XM_028260289.1 | CsATG13 | Forward | AATCAATCATACTTTTGCGTTCCCTT |
|  |  | Reverse | TCATCACTTCCTCCTCTGCCCTC |
| XM_028265144.1 | CsATG14 | Forward | AAAAGATGGGTGTAGCGGTCAGTATG |
|  |  | Reverse | TCGGATGGAATTGAATGGGGAT |
| XM_028206301.1 | CsATG16 | Forward | TGTTGCTGCTGGATCTGCTGATG |
|  |  | Reverse | TTCCAAGACCACTCCACGAACAAC |
| XM_028253213.1 | CsATG18a | Forward | TCACATCCCAACCAATCTCCCT |
|  |  | Reverse | TGTGGCGAAGCAACCGTAATC |
| [XP_028071781.1](https://www.ncbi.nlm.nih.gov/protein/XP_028071781.1?report=genbank&log$=protalign&blast_rank=1&RID=KG8BBPM6014) | CsATG18b | Forward | GCAGTGATTCGCAAGGTAGATAAA |
|  |  | Reverse | GAAAGTACCCGCTGTAGGTGATG |
| XM_028196882.1 | CsATG18c | Forward | GATGGACTTCTTCTTGCGACTGCT |
|  |  | Reverse | TCTGCCTTTTCCACTCCTCTGC |
| XM_028238387.1 | CsATG18f | Forward | GGACGGACCAACGGGCTTAT |
|  |  | Reverse | CGATGCTGCCGACTTCACA |
| XM_028202982.1 | CsATG18g | Forward | GGATAGTGGCTGTCGGTCTTGC |
|  |  | Reverse | TCTTTGGGCTTAATCGACCTGTG |
| XM_028252480.1 | CsATG18h | Forward | CTTGTTCCTGATGGCTCTGGTT |
|  |  | Reverse | CATTTTGTGACGGCATGATACG |
| XM_028265532.1 | CsATG20 | Forward | GAATGGCTACGTGATCGACTCTT |
|  |  | Reverse | TGACGCCGCATCTCAATAAA |
| XM_028236970.1 | CsATG101 | Forward | CGTGGTGCAACATTCAAAAGC |
|  |  | Reverse | CACCTTCAAGATTCGGTATGGG |
| XP_028079241.1 | CsATI | Forward | TCGAAGGATATGAGCGCCAGTA |
|  |  | Reverse | CCATCAGGAAGAAGACCAGCAGA |
| CSA033576 | CsVTI12 | Forward | CGAAGGCTACGAGCGTCAATACT |
|  |  | Reverse | CCCTCAACTTAGAAAGCAGCACAA |
| [XM_028240825.1](https://www.ncbi.nlm.nih.gov/nuccore/XM_028240825.1) | CsVTI13a | Forward | CCAAATGTAAAGGCTGTGCTGC |
|  |  | Reverse | CCTTCTACTTTCCCTAACCCTGTCA |
| XM_028226760.1 | CsVTI13b | Forward | AAGCCTGAGAAGGAAGATGAGTATGA |
|  |  | Reverse | TTAAGCTGCAAAACCTGCCACC |
| XM_028202873.1 | CsVPS15 | Forward | ACAGCGGAAGATGCCCCTACTC |
|  |  | Reverse | GAACGGTGCTCCTGAAGGTGTG |
| XM_028243914.1 | CsVPS34 | Forward | CACCTTTGGCACCAGGCGTCC |
|  |  | Reverse | AACTCCCACCATTTGCTGTCCG |
| XM_028205854.1 | CsTOR | Forward | TTTGGTGGGGCAATGACAGCAGA |
|  |  | Reverse | CTCAATGACACGCAGGCAAGCAC |
| GAAC01052498.1 | *CsPTB* | Forward | TGACCAAGCACACTCCACACTATCG |
|  |  | Reverse | TGCCCCCTTATCATCATCCACAA |
