# Supplementary material for: Genome-wide identification, characterization, and expression analysis of tea plant autophagy-related genes (CsARGs) demonstrates that they play diverse roles during development and under abiotic stress
Source: BMC Genomics. 2021 Feb 17;22:121. doi: 10.1186/s12864-021-07419-2 (PMC7891152; doi:10.1186/s12864-021-07419-2)
Supplement: Supplementary file 2 — Additional file 2: Figure S1. Motif distribution in CsATG8s subfamily members of the tea plant, Arabidorpsis, Oryza sativa, Malus domestica, Saccharomyces cerevisiae and Humans. Different motifs are represented by various colors. Figure S2. Alignment analyses of CsATI with AtATIs and OsATI. The TM-HMM-predicted transmembrane helix of CsATI (242–259) are highlighted with a yellow box. The putative N-terminal and C-terminal AIMs are highlighted with red boxes. [file 12864_2021_7419_MOESM2_ESM.docx]

Fig. S

1 Motif distribution in CsATG8s subfamily members of the tea plant, *Arabidorpsis*, *Oryza sativa*, *Malus domestica* *Saccharomyces cerevisiae* and Humans. Different motifs are represented by various colors.


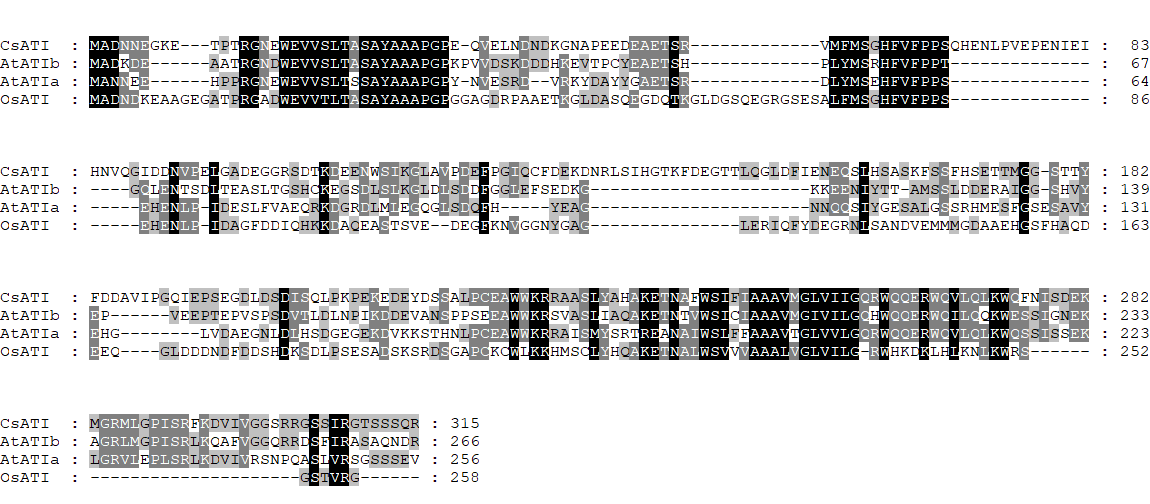


Fig. S2 Alignment analysis of CsATI with AtATIs and OsATI. The TM-HMM-predicted transmembrane helix of CsATI1 (242-259) are highlighted with a yellow box. The putative N-terminal and C-terminal AIMs are highlighted with red boxes.
